# Factors associated with use of community-based, peer-led sexual and reproductive health services by adolescent boys and young men aged 18–24 in Lusaka, Zambia: A case control study nested in the Yathu Yathu trial

**DOI:** 10.1371/journal.pgph.0002446

**Published:** 2023-11-14

**Authors:** Mwelwa M. Phiri, Bernadette Hensen, Lucheka M. Sigande, Sian Floyd, Albertus J. Schaap, Melvin Simuyaba, Lawrence Mwenge, Rosemary Zulu-Phiri, Louis Mwape, Sarah Fidler, Musonda Simwinga, Richard Hayes, Helen M. Ayles

**Affiliations:** 1 Zambart, Lusaka, Zambia; 2 Department of Public Health, Institute of Tropical Medicine, Antwerp, Belgium; 3 Department of Infectious Disease Epidemiology, London School of Hygiene and Tropical Medicine, London, United Kingdom; 4 Faculty of Medicine, Department of Infectious Disease, Imperial College, London, United Kingdom; 5 Department of Clinical Research, London School of Hygiene and Tropical Medicine, London, United Kingdom; University of Embu, KENYA

## Abstract

**Introduction:**

Adolescents and young people (AYP) aged 15–24 years have the least access to facility-based sexual and reproductive health (SRH) services, including HIV services. The Yathu-Yathu cluster-randomized trial (CRT) in Zambia tested whether a novel peer-led community-based approach increased knowledge of HIV status amongst AYP. In this nested case-control study, we aimed to identify factors associated with non-attendance to the Yathu Yathu hubs by adolescent boys and young men (ABYM) aged 18-24-years.

**Methods:**

Yathu Yathu was a CRT conducted in two communities in Lusaka, Zambia, with 10 intervention and 10 control zones. AYP in all zones were offered prevention points cards (PPC), which incentivized and tracked service use at the hubs and health facility. In intervention zones, services were provided to AYP through community-based spaces (hubs) led by peer support workers. In these zones, cases were defined as those not having accessed any service at a hub and controls as those that accessed at least one service. Data were collected from October 2020 to January 2021 and analysed using methods appropriate for unmatched case-control studies.

**Results:**

161 cases and 160 controls consented to participate in the study. Participants aged 20–24 years (adjOR 1.99, 95%CI 1.26–3.12, p = 0.003), who were educated up to college level (adjOR 8.47,95%CI 2.08–34.53, p = 0.001) or who reported being employed in the last 12 months (adjOR 2.15, 95%CI 1.31–3.53, p = 0.002) were more likely to not attend the hubs. ABYM who had a friend with a PPC were more likely to attend the hubs (adjOR 0.18 95%CI 0.09–0.35, p<0.001). Most cases reported having their last HIV test at the local government health facility (58%) while most controls reported HIV-testing at a Yathu Yathu hub (82%). Among the controls, 84% (134/160) rated the hub experience as excellent. Among cases, 65% (104/161) stated they didn’t visit the hubs “due to employment”.

**Conclusions:**

Despite Yathu Yathu services being community-based and more accessible compared to health facilities, we found age, education and employment were associated with not attending hubs. Strategies are needed to reach employed young men who may not have access to SRH/HIV services during conventional working hours and to better utilise peer networks to increase service use.

## Introduction

Across Eastern and Southern Africa, adolescents and young people (AYP) aged 15–24 years have the least access to sexual and reproductive health (SRH) services, including HIV services [1]. In a 2013 UNICEF study, only 10% of adolescent boys and young men (ABYM) and 15% of adolescent girls and young women (AGYW) aged 15–24 years reported knowing their HIV status [1]. In an analysis of survey data from the region, 29% of adolescent girls aged 15–19 reported ever testing for HIV compared to 20% of adolescent boys [2]. Despite low uptake of HIV testing and limited access to other SRH services, such as STI testing, 10.6% of ABYM aged 15–24 report having had sexual intercourse with multiple partners (≥2) in the last 12 months compared to 1.7% of AGYW [3]. Relative to adults, AYP, and ABYM in particular, are more likely to engage in higher risk sex, defined as having sex with multiple partners and low condom use, and other risk behaviours [4, 5]. A study conducted in South Africa found that men defined as “problem” drinkers based on the CAGE questionnaire (CAGE is derived from domains of the questions: Cutting down, Annoyance by criticism, Guilty feeling, and Eye-openers) [6], a substance abuse screening tool, were more likely to report having STI symptoms and one-off sexual partners compared to “non-problem” drinkers [7], linking “problem” alcohol use to increased sexual risk.

Barriers to SRH services among AYP include distance to health facilities, inconvenient operating hours and negative staff attitudes [8–10]. Access to and utilization of SRH services by ABYM is further hindered by factors such as lower knowledge of HIV/STIs, harmful masculinity norms, and mobility due to employment, with health facilities frequently seen as ‘female” spaces [4, 5, 11–14].

In Zambia, the Population-based HIV Impact Assessment (ZAMPHIA) reported an annual HIV incidence among AGYW(15-24yrs) of 0.94% compared to 0.08% among ABYM [15]. The prevalence of HIV among young women aged 20–24 years was four times greater than young men (8.3% and 2.0%, respectively). However, according to the 2018 Zambia demographic health survey, only 65.1% of young men(20-24yrs) compared to 79.2% of young women reported ever testing for HIV (3). Furthermore, a phylogenetics study conducted as part of the HPTN 071 (PopART) trial of universal testing and treatment (UTT) found that men aged 25–34 years are the age group which contributed most to onward transmission of HIV, despite a lower incidence and prevalence of HIV relative to their female peers [16]. With HIV testing being critical to epidemic control, facilitating access to treatment and prevention services and considering results from ZAMPHIA and phylogenetic studies, men should be prioritized for interventions to achieve UTT and improve access to prevention services [15, 16].

In regions such as eastern and southern Africa, which have the highest burden of HIV globally, community-based provision of SRH services, including HIV services, has been suggested as a solution to closing the knowledge, SRH service use and HIV continuum gaps for men, as well as influencing changes in health-related attitudes and behaviours [14, 17–19]. Currently, services are primarily available in established healthcare facilities. Experience in Zambia with the home-based PopART intervention shows that, while successful in reaching a large proportion of participants with UTT, gaps were revealed in HIV testing and ART coverage among ABYM aged 18–24 years. This age group also took the longest time to link to HIV care [20–22]. The implementation of community-based hubs, (spaces) which were away from the health facilities, in PopART communities after the end of the HPTN 071 (PopART) trial reached a high number of young men [19] suggesting a community-based hub approach could effectively reach men.

Yathu Yathu was a cluster-randomised trial (CRT), which included provision of community-based and peer-led SRH services, including HIV testing, through spaces called Yathu Yathu hubs [23]. Co-designed with AYP, including ABYM [24], the intervention was expected to be youth-friendly and to enhance AYP’s use of SRH services. With evidence that men are less likely to access healthcare services, this nested case-control study aimed to understand reach among ABYM, identify what factors were associated with non-attendance to the Yathu Yathu hubs and what hub characteristics appealed to ABYM accessing SRH services from the hubs to inform implementation of community-based services to achieve high reach among ABYM.

## Methods

### Study population and location

Yathu Yathu (“For us, by us”) was conducted in two high density urban communities in Lusaka, Zambia, between September 2019 and September 2021. Details of the CRT are described elsewhere [23]. Briefly, the two communities were each split into ten geographical zones (clusters). Each community had five zones randomised to receive the intervention and five zones randomised to standard-of-care. Each zone had a population of approximately 2,350 adolescents and young people aged 15–24 years.

#### The Yathu Yathu intervention

The Yathu Yathu intervention had two key components, (i) spaces called Yathu Yathu hubs and (ii) Prevention Points Cards (PPC). Yathu Yathu hubs were located away from the health facility in each community and provided peer-led SRH services, such as HIV testing, STI screening, condom distribution and comprehensive sexuality education. The PPC were plastic cards provided to all AYP who consented (with assent and written parental/guardian consent if aged <18years old) to participate in the study during a census conducted at the start of the study (August 2019-January 2020). The PPC was part of the PPC ‘system’, which included provision of ‘rewards’. Rewards were health and non-health related products, such as bathing soap, toothpaste, exercise books and pens. AYP gained points for using services, which they redeemed to receive these rewards. The PPC also allowed the study team to track who accessed services, what services were accessed and what rewards were “purchased” using accumulated points [23].

#### Case-control study

For this nested case-control study, cases were defined as ABYM who received a PPC but did not attend the Yathu Yathu hub to access any services since the start of implementation of the Yathu Yathu intervention (September 2019). Controls were defined as ABYM who received a PPC and accessed at least one service at a Yathu Yathu hub since January 2020 after the census and PPC distribution were complete. Participants were drawn from the ten intervention zones that were randomly allocated to the intervention arm. Eligible participants were ABYM aged 18–24 years at the time of receiving the PPC, who had been enumerated and consented to participate in Yathu Yathu, accepted a PPC and were willing and able to provide consent. Adolescent boys aged 15–17 years were not included due to early monitoring of data on access showing double the attendance for services compared to those 18-24years old [25].

A list of eligible individuals who fulfilled the definition of being a case or control was generated from the census database. We used simple random sampling, stratified by community, to randomly select a list of cases and controls to invite for participation in the study. For controls, we randomly selected an equal number of ABYM who had been to the hub once and ABYM who had visited the hub on multiple occasions.

### Data collection

Data were collected from October 2020 to January 2021, to understand who was accessing services within the first full year of implementation (January 2020-January 2021). Participants were visited at their household for initial contact. Two attempts for face-to-face interviews at the household were made. If unable to conduct face-to-face interviews at the household, phone interviews or face-to-face interviews at a place of the participant’s choice, were conducted. Phone interviews were introduced in response to the COVID-19 pandemic. Visit attempts to collect data were conducted in the order generated by the computer until the sample size was reached.

The questionnaire, administered by a male research assistant, included modules on: socio-demographics, such as age, marital status, highest level of education attained, employment and living conditions; household characteristics, including household assets, how many men they lived with, the number of people they lived with and relationship with the men lived with; intrapersonal factors, including peer pressure using a scale developed and validated to measure peer pressure [26], and reproductive health service use questions of the Gender Equitable Men’s (GEM) scale [27]. The questionnaire also included the AUDIT scale to measure harmful alcohol use [28], the SRQ-10 scale to measure mental health [29] and questions on sexual behaviours. Inclusion of these domains was guided by literature on barriers to SRH service use by men in Eastern and Southern African countries and by factors hypothesized to influence service use at the hubs.

### Sample size calculations

Using an unmatched study design, the sample size was estimated as ~160 cases and ~160 controls, with a total sample size of approximately 320 ABYM. This sample size was calculated by assuming that the percentage of controls exposed to particular risk factors, for example at risk of alcohol use disorders (which has been identified as a risk factor potentially affecting HIV service use [30]), was 25%, 30% or 35%, and that the odds ratio comparing ABYM exposed with individuals unexposed was 2.0; the study had >80% power to detect these associations (with p<0.05).

### Data analysis

All variables were described by case/control status, including age, marital status, influence of friends on access to services, sexual behaviours, such as number of sexual partners in last 12 months, use of condoms in last 12 months, and age of last sexual partner. For controls only, we described experiences of hubs and for cases only, we described reasons for non-attendance.

Age was characterised into two levels, 18–19 and 20–24 years old; education level on three levels, primary school, secondary school and tertiary, based on the current school grading system; socioeconomic status, using Principle Components Analysis(PCA), was categorised into three quintiles due to the small sample size and based on building, flooring, drinking water, toilet type, energy source and assets. Peer pressure was scored with > = 12 categorised as susceptible to peer pressure and <12 as not susceptible to peer pressure; GEM score > = 18 supporting equitable gender norms and <18 supporting inequitable gender norms; AUDIT score > = 8 categorised as harmful alcohol use and <8 as not having harmful alcohol use; those who had experienced mental health distress having a score > = 7/20 and <7/20 for those without.

**Fig 1:**
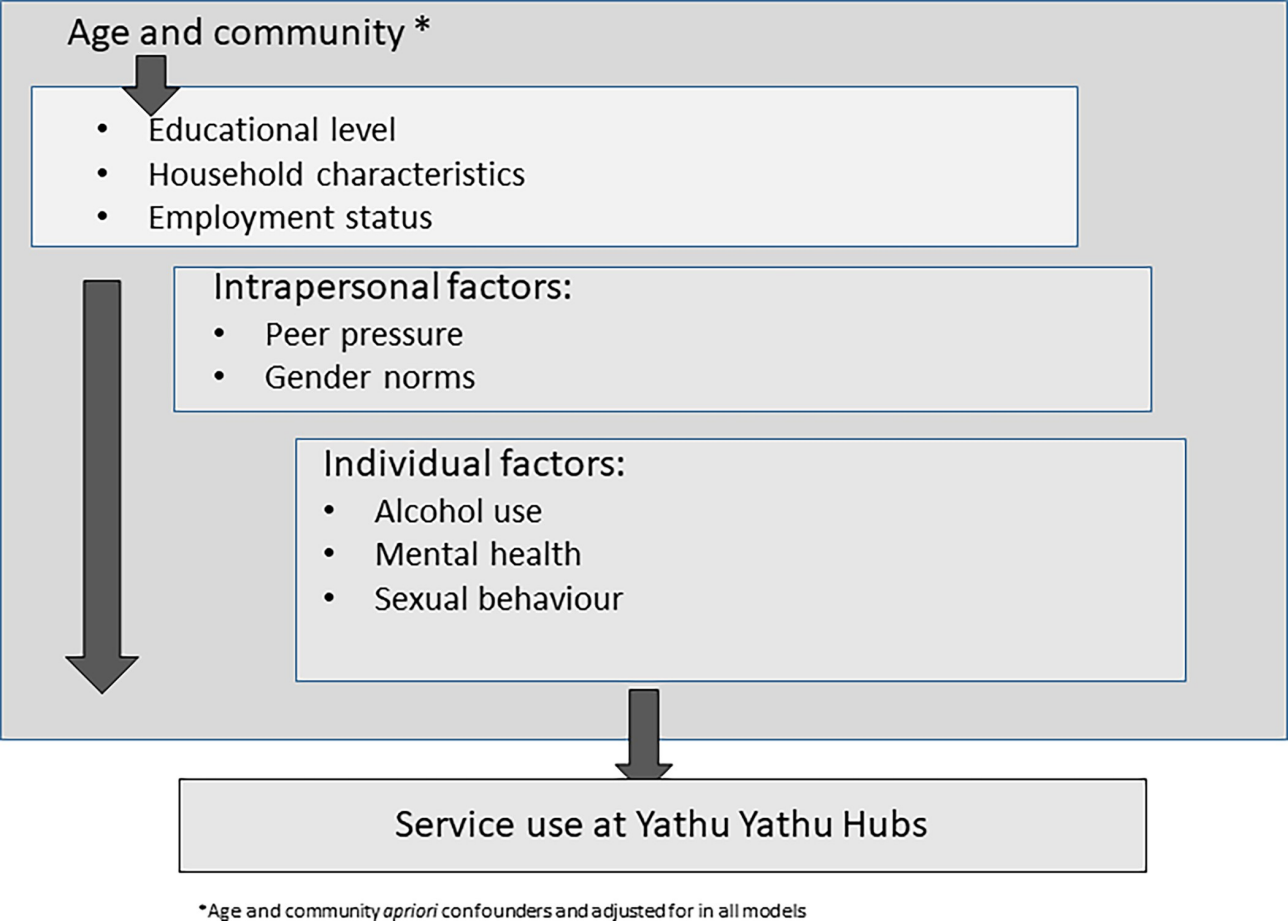
Conceptual framework for exploring the association between variables of interest and not accessing services at Yathu Yathu hubs.

Data were analysed using methods appropriate for the analysis of unmatched case-control studies. Multivariable logistic regression was used to test for associations between the factors of interest and being a case, adjusting for potential confounders as described above. Age was considered an *a priori* potential confounder and community was adjusted for to account for potential clustering by community. For the analysis of factors associated with not attending a hub, a conceptual framework was developed to guide the analysis (Fig 1) such that, in adjusted analyses, we used the hierarchical approach, as described by Victora et al, in which variables higher in the framework influenced the variables below them [31]. If variables in an upper level were found to be associated with being a case, variables in levels below this variable in Fig 1 were adjusted for these variables but not vice versa. Variables associated with the outcome (p<0.1) in univariable analysis were included in multivariable models. Likelihood Ratio Tests (LRT) were used to assess statistical evidence of association. Adjusted ORs (adjOR), 95% CIs and LRT p-values were presented.

### Ethical considerations

Ethical approval was obtained from the University of Zambia Biomedical Research Ethics committee (REF: 007-04-19) and the London School of Hygiene and Tropical Medicine Ethics committee (REF: 17104 ‑ 04). All participants were asked to provide written informed consent. At the time of data collection, face-to-face interviews were not always feasible due to COVID-19-related restrictions. In response, ethical approval was obtained from both committees to conduct phone interviews with verbal informed consent. Verbal informed consent was recorded and stored on a password-protected computer.

## Results

**Fig 2:**
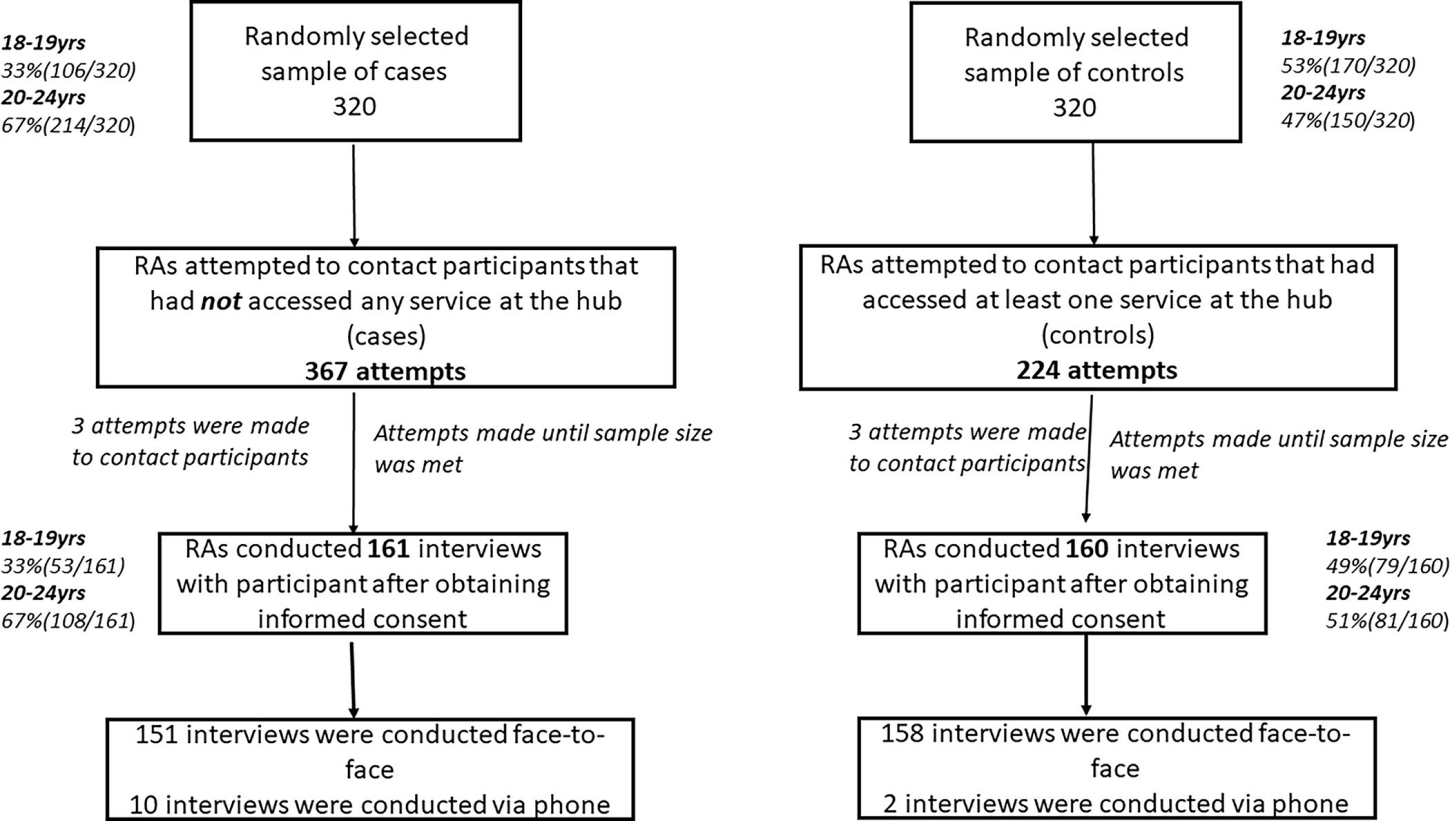
Cases (non-attendance of SRH services at hubs) and controls (attendance of at least one SRH service at hubs) selection process.

Overall, 640 ABYM (320 cases and 320 controls) were randomly sampled. A total of 606 visit attempts (including multiple attempts for some) were made to contact ABYM. For cases, 366 visit attempts were made until sample size was reached compared to 225 visit attempts for controls. This resulted in 161 cases and 160 control participants being interviewed (Fig 2).

Comparing the age strata of the cases and controls randomly selected to those reached, the proportions were similar (Fig 2). Among cases who could not be contacted/declined/not approached for interviews (N = 159), and based on enumeration data collected during the census, there were differences in education level: with 83% (132/159) reporting secondary school education compared to 77%(124/161) among those reached.

### Socio-demographic, household conditions, lifestyle and health characteristics

Young men aged 20–24 years were more likely not to have attended hubs compared to those aged 18–19 years (67% 108/161 vs 51% 89/160; OR 1.99, 95%CI 1.26–3.12, p = 0.003). Most participants reported having completed secondary education (Table 1). Participants with an education level up to college/university/trade school were more likely not to have attended hubs compared to those with only primary or secondary school education (adjOR 8.47,95%CI 2.08–34.53, p = 0.001). There was evidence that participants currently in school were more likely to have attended hubs (adjOR 0.60,95%CI 0.37–0.98, p = 0.04). Participants who were employed in the last 12 months were twice as likely to not have attended hubs compared to those not employed (adjOR 2.15, 95%CI 1.31–3.53, p = 0.002). ABYM who knew a friend with a PPC were more likely to have attended hubs compared to those who did not (adjOR 0.18 95%CI 0.09–0.35, p<0.001) (Table 1).

**Table 1:** Socio-demographic, household conditions, lifestyle, and health characteristics.

Variables	Cases (col%)	Controls (col%)	OR*	LRT- p-value	adjusted OR (95%CI)**	LRT p-value
** **	**161**	**160**	** **	** **	** **	** **
**Age**						
18–19	53 (33%)	79(49%)	1	**0.003**	1	**0.003**
20–24	108(67%)	81 (51%)	1.99 (1.26–3.12)		1.99 (1.26–3.12)	
**Education level** *						
primary school (grade 0–7)	17(11%)	21 (13%)	1	**0.002**	1	**0.001**
secondary school (grade 8–12)	124(77%)	136 (85%)	1.09 (0.54–2.18)		1.20(0.59–2.43)	
college/university/trade school	20 (12%)	3 (2%)	7.15(1.79–28.56)		8.47(2.08–34.53)	
**Currently in school** *	** **	** **				
NOT currently in school	85 (53%)	64 (40%)	1	0.04	1	**0.04**
Currently in school	76 (47%)	96 (60%)	0.63(0.39–0.99)		0.60(0.37–0.98)	
**Marital status** *	** **	** **				
NOT married	152 (94%)	152 (95%)	1	0.79	1	0.79
married	9 (6%)	8 (5%)	0.87(0.32–2.37)		0.87(0.31–2.42)	
**Employment status in last 12 months** *	** **	** **				
Unemployed	47 (29%)	79 (49%)	1	**0.003**	1	**0.002**
Employed	114 (71%)	81 (51%)	2.06(1.28–3.33)		2.15(1.31–3.53)	
**Number of men≥ 18 living with** *						
None or 1 only	74(46%)	81 (51%)	1	0.53	1	0.48
2–3 men	69(43%)	62 (39%)	1.32(0.82–2.12)		1.35(0.82–2.22)	
≥4	18(11%)	17(10%)	1.20(0.572.51)		1.05(0.48–2.34)	
**Having a friend with a PPC** **						
No	48(30%)	16(10%)	1		1	**<0.001**
Yes	76(47%)	138(86%)	0.19(0.10–0.37)	0.00	0.18(0.09–0.35)	
I don’t know	37(23%)	6(4%)	2.09(0.74–5.9)		2.14(0.75–6.31)	
**Peer Pressure** **						
no peer pressure score ≤11	122(76%)	116(72%)	1	0.37	1	0.49
Peer Pressure score ≥12	39(24%)	44(28%)	0.79(0.47–1.32)		0.82(0.48–1.43)	
**AUDIT score** ***	** **	** **				
Audit score ≤ 7	131 (81%)	138(86%)	1	0.64	1	0.68
Audit score ≥ 8	30 (19%)	22(14%)	1.17(0.61–2.24)		1.17(0.56–2.46)	
**Wealth Index (3 categories)** *						
1 (low)	51(32%)	56(35%)	1	0.77	1	0.74
2 (medium)	55(34%)	52(32.5%)	1.20(0.69–2.08)		1.15(0.66–2.03)	
3 (high)	55(34%)	52(32.5%)	1.18(0.68–2.04)		0.92(0.51–1.66)	
**Mental Health** ***(depression, anxiety-related disorders and somatoform disorders)	** **	** **				
Self-reporting questionnaire-10 score ≤6	147 (91%)	149 (93%)	1	0.58	1	0.94
Self-reporting questionnaire-10 score ≥7	14 (9%)	11 (7%)	1.26(0.54–2.92)		1.04(0.39–2.75)	
**Reproductive health gender norms** **						
inequitable norms score ≤17	72(45%)	74(46%)	1	0.93	1	0.84
equitable norms score ≥18	89(55%)	86(54%)	1.02(0.65–1.59)		0.95(0.60–1.52)	

*adjusted for age and community, apriori

** adjusted for age, community, education level, employment status in last 12 months

*** adjusted for age, community, education level, employment status in last 12 months and having a friend with a PPC card based on hierarchical approach described in Fig 1

Cases defined as ABYM who received a PPC but did not access any services from a Yathu Yathu hub since the start of implementation of the Yathu Yathu intervention (September 2019)

Controls defined as ABYM who received a PPC and accessed at least one service at a Yathu Yathu hub since January 2020 after the census and card provision was completed

There was no evidence for an association between other factors and not accessing services, including being married (adjOR 0.87; 95%CI 0.31–2.42, p = 0.79), susceptible to peer pressure (adjOR 0.82,(95%CI 0.48–1.43,p = 0.49), inequitable reproductive health gender norms (adjOR 0.95,95%CI 0.60–1.52, p = 0.84), at risk of harmful alcohol use (adjOR 1.17,95%CI 0.56–2.46, p = 0.68) and experiencing mental health distress (adjOR 1.04,95%CI 0.39–2.75, p = 0.94) (Table 1).

### Sexual behaviour characteristics and use of SRH services

Among cases and controls, 76% (243/321) reported ever having had sex. There was weak evidence that ABYM who had more than one lifetime partner had greater odds of not accessing any service at the hubs (adjOR1.89, 95%CI 0.81–4.44,p = 0.1) (Table 2). Other sexual behaviour characteristics such as the age of sexual debut, whether they used a condom with their last sexual partner, alcohol use at last sexual encounter and age of last sexual partner, were not associated with hub attendance.

**Table 2:** Sexual behavior characteristics and use of SRH services of cases and controls***.

	Cases	Controls	OR*	LRT p-value	Adjusted OR**	LRT p-value
	**161**	**160**				
**Have you ever had sex (vaginal or anal penetrative sexual intercourse)**						
No	20% (32/161)	29% (46/160)	1	0.36	1	0.15
yes	80% (129/161)	71% (114/160)	1.29 (0.74–2.24)		1.61(0.84–3.08)	
**Age at first sex**						
< = 14 years old	18% (23/129	14% (16/114)	1	0.34	1	0.61
15–19 years old	62%(80/129)	75%(85/114)	0.73(0.35–1.50)		0.67(0.30–1.49)	
> = 20 years old	20% (26/129)	11%(13/114)	1.23(0.48–3.14)		0.77(0.26–2.29)	
**Number of lifetime sexual partners**						
1 partner	19% (24/129)	23% (26/114)	1	0.06	1	0.10
2–4 partners	44% (57/129)	56% (64/114)	0.90(0.46–1.77)		0.91(0.43–1.95)	
> = 5 partners	37% (48/129)	21% (24/114)	1.87(0.87–4.00)		1.89(0.81–4.44)	
**Condom use with last sexual partner**						
No	50% (65/129)	39% (45/114)	1	0.16	1	0.76
Yes	50% (64/129)	61% (69/114)	0.69(0.41–1.15)		0.91(0.49–1.67)	
**Age of last sexual partner**						
≤14 years old	2.3% (3/129)	2.6% (3/114)	1	0.38	1	0.64
15-19years old	59.7% (77/129)	71.9% (82/114)	0.66(0.12–3.49)		0.74(0.11–4.77)	
≥20years old	38.0% (49/129)	25.4% (29/114)	0.97(0.17–5.51)		1.00(0.14–6.98)	
**Did you drink alcohol the last time you had sex with your last partner**						
No	85% (109/129)	89% (102/114)	1	0.33	1	0.21
Yes	15% (20/129)	11% (12/114)	1.47(0.67–3.21)		1.76(0.71–4.35)	

*adjusted for age and community apriori

** adjusted for age, community, education level, employment status in last 12 months and having a friend with a PPC card based on hierarchical approach described in Fig 1

***only includes data from those that reported as having had sexual intercourse before except for variable “ever had sex”

Cases defined as ABYM who received a PPC but did not access any services from a Yathu Yathu hub since the start of implementation of the Yathu Yathu intervention (September 2019)Controls defined as ABYM who received a PPC and accessed at least one service at a Yathu Yathu hub since January 2020 after the census and card provision was completed

Among controls who reported having had sex before, 88% (137/155) had an HIV test in the last 12 months compared to 49% (69/142) of cases. In addition, 24% (34/142) of cases had used an HIVST before compared to 37% (58/155) of controls. Most cases reported having their last HIV test at the local government health facility,58% (83/142) while 82% (127/155) controls reported Yathu Yathu hubs. When asked about their source of condoms in the last 12 months, 37% (51/161) of cases reported government facilities as their most common source, while 73% (117/160) of controls reported Yathu Yathu hubs as their most common source of condoms (Table 3).

**Table 3:** Location of SRH services accessed by cases and controls.

	Cases	Controls
	**161**	**160**
**Location of last HIV test**		
Government hospital/clinic	58% (83/142)	10% (16/155)
Mobile testing tents	36% (51/142)	6% (10/155)
Yathu Yathu hubs	-	82% (127/155)
Other (private/mission hospital/Pop ART hubs)	6% (8/142)	2% (2/155)
**Location of last screening/testing for STI**		
Government hospital/clinic	94% (17/18)	20% (9/44)
Mobile testing tents	-	-
Yathu Yathu hubs	-	73% (32/44)
Other (private/mission hospital/Poupart hubs)	6% (1/18)	7% (3/44)
**Source of condoms in last 12 months**		
I did not collect any condoms anywhere	19% (30/161)	8% (13/160)
Government hospital/clinic	37% (59/161)	6% (10/160)
Mobile testing tents	2% (3/161)	-
Yathu Yathu hubs	1% (2/161)	73% (117/160)
Private pharmacy	5% (8/161)	2% (3/160)
Kantembas(small private shop)	13%(21/161)	<1%(1/160)
Other(private/mission hospital/PopART hubs)	24%(38/161)	10%(16/160)

### ABYM’s perceptions of hubs and the hub characteristics

Among controls, 47% (75/160) visited the hub once, 14% (23/160) twice and 39% (62/160) three times or more. In addition, 45% (36/79) of the 18–19 years old reported visiting more than 3 times compared with 32% (26/81) of the 20-24year olds. When asked about their first experience visiting the hubs, 84% (134/160) rated this as ‘excellent’, 10% (16/160) as ‘above average’ and 6% (10/160) as ‘average’. This distribution was similar by age group. None of the participants rated it as ‘poor’ or ‘very poor’.

Almost half (46% n = 75/160) of the controls reported visiting a hub for the first time because they were encouraged by a Yathu Yathu staff member, while 30% (n = 49/160) visited because they were curious about the hubs and 16% (26/160) because a friend encouraged them.

**Fig 3:**
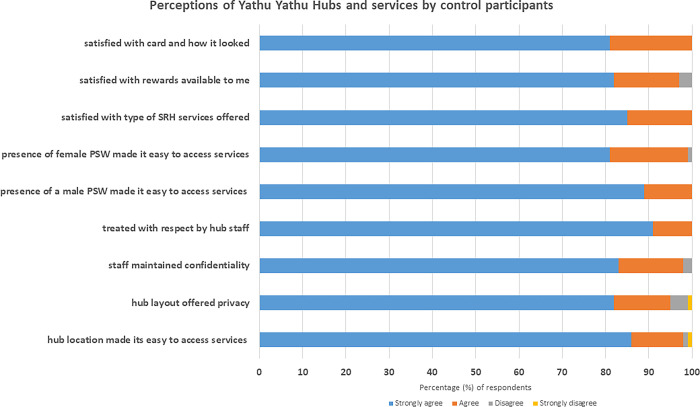
Perceptions of Yathu Yathu hubs and services by control participants.

The majority (>80%) of controls strongly agreed that the hubs were suitable and accessible locations, were satisfied with the type of SRH services offered, were treated with respect by hub staff and confident that the staff maintained their privacy and confidentiality. However, a small percentage (5%; n = 8/160) disagreed/strongly disagreed that the hub they accessed offered adequate privacy; and 3% (4/160) disagreed with the statement that the “rewards offered were satisfactory to me” (Fig 3).

**Fig 4:**
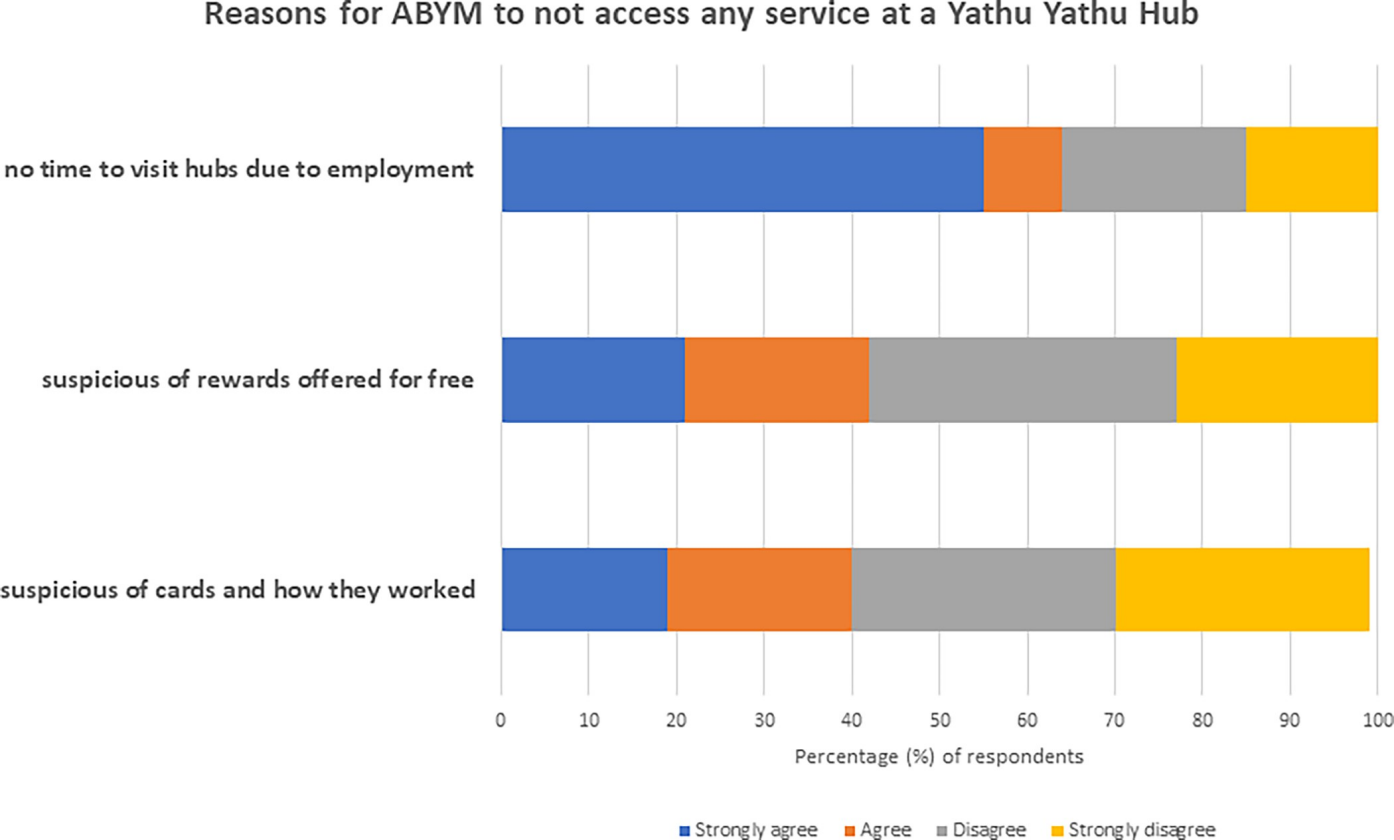
Reasons for ABYM to not access any service at a Yathu Yathu hub.

Among the cases, 65% (104/161) strongly agreed/agreed with the statement “I did not have time to visit (hubs) because of my employment”. Less than 25% strongly agreed or agreed that one of the reasons for not visiting the hubs was due to being suspicious of the cards or the rewards (Fig 4). The other most commonly reported reason for not accessing any service at the hubs was “being busy” (12%; n = 19/161).

## Discussion

In this nested case-control study, we found that age, level of education and employment status were associated with *not* attending Yathu Yathu hubs to access SRH services. In addition, we found that knowing a friend who had a PPC was associated with service access. The majority of ABYM who visited a hub at least once expressed positive perceptions about the hubs, with more than a third accessing services at the hubs three times or more. This suggests that the Yathu Yathu model of peer-led SRH services can reach ABYM, particularly those that are in school and less than 20 years old. However, for young men aged 20–24 years and ABYM who are in college or in employment, greater efforts and strategies that address barriers to service access are required to reach them with SRH services.

The primary outcome of the Yathu Yathu CRT (self-reported knowledge of HIV status), revealed that overall,73% of AYP in the intervention arm knew their HIV status compared to 48% in the control arm and showed that the intervention had a greater effect among adolescent boys aged 18–19 [32]. This is reflected in this sub-study, where we saw that this age group was more likely to access services than the 20–24-year-olds. This could suggest that the hubs were spaces that were acceptable for SRH service access especially among the adolescent boys, who would generally be unemployed and therefore be more available to access services or may indicate that those >20 years do not necessarily want to access services with <20 years as noted after COVID-19 adaptations [33]. This is consistent with other community-based interventions reaching men and young men in particular, with HIV testing services [18, 19, 34].

In this study, controls were more likely to have a friend who had a PPC than cases, suggesting that having friends or knowing someone who has accessed SRH services can encourage service use among ABYM. The influence of peers in accessing SRH services such as condoms and HTS has been reported elsewhere [35, 36], with young men observed to come in groups to access HTS services during the provision of community-based HTS services implemented after the PopART study [19]. However, a systematic review on condom use among adolescents in a study conducted in southern Africa, suggested peers could be a barrier to condom use, especially among adolescent boys, thus suggesting a negative peer influence on SRH-related behaviours [37]. On the other hand, despite almost a quarter of all study participants reporting being susceptible to peer pressure, we found no evidence of an association with not accessing services at hubs. This may suggest that while peers or other men may negatively influence sexual behaviours such as condom use, they could also positively influence access to HIV services, as was found in studies in Tanzania [35, 38] and Zambia [39]. In addition, while most sexual behaviours in our study were not associated with accessing hub services, a higher percentage of ABYM who attended hubs tested for HIV and screened for STIs than those who didn’t attend hubs, which could have been a result of accessing Yathu Yathu services.

Community-based (e.g. door-to-door HTS and mobile HTS) provision of services for HIV testing and other SRH services has been suggested to reach men and has proven successful in various countries including Zambia, Zimbabwe and South Africa [14, 17–19, 40]. This finding is consistent with the results of the present study, which found that more than 80% of ABYM that accessed the hubs considered them satisfactory in terms of services provided, the providers and service location. However, as has been reported in other studies exploring strategies to provide SRH services to men [19, 21], young men aged 20–24 years, did not access services as much as adolescents aged 18–19 years. The most common reason cited for non-attendance was being busy or at work/school. Other studies have suggested providing services in the evening, having male-focused clinics and/or providing HTS services at workplaces [14, 41, 42]. In Yathu Yathu, services were provided during normal hours (08:00hrs-16:30hrs); however, services were also provided every Saturday and on alternate Sundays to accommodate AYP who were in school or working, this may suggest alternative reasons, which were not included in the questionnaire, such as not wanting to access services with younger age groups. However, the limited opening hours could explain why young men were unable to access services, particularly if they were working or busy with college. In spite of this, overall, a high number of ABYM who accepted a PPC accessed services in the Yathu Yathu hubs compared to the control arm [43] even in the context of the COVID-19 pandemic (i.e. reduction in number of ABYM accessing services due to adaptations for infection control) [33] and implies the Yathu Yathu community based model can successfully reach ABYM.

Inequitable gender norms have been linked to negative SRH outcomes, such as having multiple partners and gender-based violence and thus higher risk of HIV infection, with men more likely to express inequitable gender norms compared to women [14, 44]. In this study, we only looked at the effect of gender equitable norms on reproductive health on accessing SRH services, we found that almost half of all men had inequitable norms but found no association between equitable reproductive health norms and accessing services at the hubs and may suggest other barriers such as employment are more important [11]. Nonetheless, hubs may provide an opportunity to address and challenge any gender inequitable norms among ABYM particularly on encouraging uptake of HIV services [35] and gender-based violence towards women [44].

Studies have shown that harmful alcohol use is associated with poor SRH outcomes, such as not using a condom, having multiple partners and inhibiting access to HIV testing for some populations [7, 45]. Considering this evidence, we explored whether being at risk of alcohol misuse was associated with not accessing services at the hubs. We found no statistical evidence to support this in this study. This is similar to a study assessing alcohol consumption as a barrier to HIV testing in Uganda, which found it was not a barrier in men but was a potential barrier among women [45].This finding highlights a gap in research on whether being at risk of alcohol misuse is actually a barrier to use of SRH services by ABYM or whether it is the way services are provided not meeting the needs of those at risk of alcohol use [38, 46, 47].

Our study has limitations. Due to high mobility of ABYM in these study communities, which has been reported elsewhere [11, 19], it was difficult to find and contact participants that were cases compared to controls. Reaching the controls may have been easier because they had already engaged with the hubs. This may have introduced selection bias towards those easier to contact resulting in an overestimation of associations with exposures. This was mitigated by ensuring that a similar number of attempts were made to contact and enroll participants among cases and controls. Another limitation could be social desirability bias, especially among controls, in their responses regarding their experiences at the hubs and sexual and alcohol use behaviours which were part of sessions at the hubs. Lastly, observer bias by the research assistants could have been possible, as we could not blind research assistants to who was a case and who was a control. This is because experiences of controls required questions on the experiences of hubs to be included. However, this was mitigated by conducting interviews with controls away from the hubs.

Despite limitations, the study covered a range of factors that have been hypothesized to affect SRH service use among young men and contributes to the limited evidence available for this population. It provides important information on what components of the implementation design could have improved uptake of services especially among young men aged 20–24 years, such as evening hours and/or offering services at places the young men are found and suggests that peers can influence accessing SRH services.

## Conclusions

This nested case-control study provides important insights into factors that influence ABYM access to community-based peer-led SRH services. It provides evidence that the hubs, as designed, were largely acceptable and accessible to ABYM even during the COVID-19 pandemic, but that if ABYM were older, working or in college, this impacted their ability to access SRH services at the hubs. This implies more needs to be done to address structural barriers to reach young men aged 20–24 years with community-based SRH services. This may include working outside ‘normal’ hours during the week (and not just weekends) and considering providing Yathu Yathu services at workplaces or busy places such as bus stations where men are found to ensure access among ABYM as well as leveraging peer networks to provide positive influences and facilitate service use.
